# Beyond IQ: Systemic Resources in STEM Achievement

**DOI:** 10.3390/jintelligence14030045

**Published:** 2026-03-11

**Authors:** Albert Ziegler, Sonja Bayer, Heidrun Stoeger

**Affiliations:** 1 Chair of Educational Psychology and Research on Excellence, Friedrich-Alexander University Erlangen-Nuremberg, 90478 Erlangen, Germany; albert.ziegler@fau.de; 2 Department of Educational Sciences, University of Regensburg, 93053 Regensburg, Germany; sonja.bayer@ur.de

**Keywords:** intelligence, Actiotope model, educational capital, learning capital, STEM achievement, incremental validity, systemic identification

## Abstract

There is a growing consensus that we must look beyond IQ to understand the mechanisms of talent development. Grounded in the Actiotope Model of Giftedness, this study adopts a resource-based approach and examines the incremental and interactive contributions of educational and learning capital to STEM achievement beyond IQ. Data were collected from 318 German secondary school students (grades 6–10; *M*_age_ = 12.08; 50.3% male) using domain-specific measures of educational and learning capital, a nonverbal matrix intelligence test, and STEM grades. Robust regression and mediation analyses showed that learning capital significantly predicted STEM achievement beyond general intelligence, whereas educational capital exerted no direct effect. Instead, the relationship between educational capital and achievement was fully mediated by learning capital. Moreover, the interaction term of educational and learning capital predicted achievement. A further interaction indicated that the positive effect of learning capital on STEM achievement was stronger for students with higher intelligence, consistent with an intelligence utilization (Matthew) effect. These findings support a systemic interpretation of achievement in which intelligence reflects prior resource utilization and functions as a catalyst, while current learning resources constitute the proximal determinants of STEM performance.

## 1. Introduction

For decades, the field of gifted education has been dominated by a clear, albeit narrow, paradigm: equating high ability with high general intelligence (Callahan, 2000). This view has come under heavy criticism for several reasons. Modern concepts of giftedness have therefore abandoned this limited understanding of giftedness (for an overview, see Dai, 2023; Sternberg, 2018; Sternberg & Ambrose, 2021). As highlighted by this Special Issue, there is a growing consensus that we must look beyond IQ to understand the mechanisms of talent development. Often, this search leads to the inclusion of additional personality traits or competencies. Examples include Emotional Intelligence (EQ) or Creativity (CQ; Matthews et al., 2018; Sternberg, 2005; Treffinger, 1986). While valuable, adding more traits to the list does not necessarily explain the process of talent development. Therefore, in this study, we propose a more fundamental paradigm shift: moving from a trait-based perspective to a systemic resource-based perspective. We argue that to understand who excels, we must look at the educational and learning capital available to learners.

### 1.1. Theoretical Framework

Our theoretical approach is the Actiotope Model of Giftedness (AMG; Ziegler, 2005; Ziegler & Stoeger, 2017b). An actiotope encompasses a person and their immediate environment with which they interact actively. For example, two siblings may operate in the same environment but have completely different actiotopes because they interact with different parts of that environment. In contrast to trait-based theories, this systemic approach views talent development as an adaptation process resulting from the interaction between an individual and their environment (see also Csikszentmihalyi, 1998; Dai, 2009). The model focuses on the accumulation and effective use of resources.

The AMG distinguishes between two types of resources: exogenous resources, i.e., resources in a person’s environment (also referred to as educational capital), and endogenous resources, i.e., resources within a person (also referred to as learning capital). These capitals are those resources within the person and their environment that can be used for development and learning. Its focus on development and learning distinguishes the approach from other uses of the term capital. For example, according to Bourdieu (1986), social capital refers to resources that a person mobilizes to maintain or improve their social position.

Educational capital comprises five forms: economic, social, infrastructural, cultural, and didactic educational capital (for an overview, see Ziegler et al., 2019). Economic educational capital refers to financial and material resources and represents a special case. As a proto capital, it cannot directly promote development and learning but must first be converted into one of the other types. Social educational capital refers to social resources that support an individual’s development. Examples include parents, teachers, and mentors. Infrastructural educational capital refers to the material and informational environment conducive to learning, including books and quiet study spaces. Cultural educational capital encompasses values, norms, and learning climates that foster development, e.g., an error culture and educational goals anchored in curricula. Finally, didactic educational capital refers to the know-how for designing and improving educational and learning processes, such as well-trained teachers, effective curricula, and support for professional talent development.

Learning capital also comprises five forms: organismic, telic, actional, episodic, and attentional capital. Organismic learning capital also serves as a proto resource. It encompasses all physiological processes that influence educational outcomes, including physical fitness, health, and nutrition. Like economic capital, it must be transformed into other forms to be effective. Telic learning capital comprises a person’s “evaluative machinery”, including their goal-directedness, values, and anticipated target states. Actional learning capital refers to an individual’s action repertoire—specifically, the knowledge and skills available to solve tasks. Episodic learning capital consists of patterns of experience that enable the simultaneous comparison of current situations with past ones, facilitating intuitive decision-making. Finally, attentional learning capital refers to the quantitative and qualitative attentional resources a student can allocate to the learning process.

The value of the ten capitals depends on their coordination (Ziegler & Stoeger, 2017b). For example, a library (infrastructural capital) or a mentor (social capital) remains inert unless the student utilizes endogenous resources to interact with them and leverages them effectively to improve learning. In this sense, educational capital must be transformed into learning capital to act as a functional resource for achievement.

The absence of any vital resource limits development (Salisbury, 1992). This law of the minimum also applies to talent development (Naujoks-Schober et al., 2025). Even high intelligence cannot compensate for a lack of vital resources such as motivation or social support. Consequently, talent development is not the result of a single trait, such as IQ, but the outcome of a system.

In the AMG, general intelligence (*g*) is not viewed as a fixed biological constant or mere processing speed. Instead, we interpret it as a result of the past. Intelligence tests measure problem-solving and academic skills (Ziegler & Stoeger, 2017b). A high score, therefore, reflects a highly effective action repertoire (actional learning capital) built up to that point. It shows that the student has successfully transformed educational opportunities into skills in the past.

However, IQ only looks backward. It represents a history of successful resource use, but it does not guarantee the future. For further talent development, the necessary resources must still be available. This is why measuring current educational and learning capitals is essential. This perspective also explains why we expect an interaction between IQ and available capital. Students with high IQs have proven they can use resources effectively to build their action repertoire. Therefore, we expect them to profit more from the capital available to them now. This leads to a “Matthew Effect”: those who successfully used resources in the past are better equipped to use them in the present.

### 1.2. The Present Study

The aim of our study is to investigate the discussed relationships between IQ and capital. Specifically, we want to uncover the interactive and incremental contributions of educational and learning capital to STEM achievement beyond IQ. We chose the domain of STEM (Science, Technology, Engineering, and Mathematics) because it traditionally is viewed through a strong “talent” lens (McCabe et al., 2020). In addition, STEM learning is highly cumulative. Missing a resource often creates a bottleneck that cannot be bypassed (Ziegler & Stoeger, 2017b). By demonstrating the predictive power of systemic resources in this domain, we provide a robust test for the validity of the resource-based approach. Based on our theoretical framework, we derived four hypotheses.

**Hypothesis** **1.** *Incremental Validity Hypothesis.* Learning capital in STEM makes a unique contribution to predicting STEM achievement beyond general intelligence.

We defined intelligence not as a biological constant, but as a measure of the effectiveness of the action repertoire built in the past, or in other words, a person’s history of success. However, solving current problems in STEM also requires currently available resources. A high IQ indicates that a student could build these resources efficiently, but it does not replace them. Because educational capital exerts its influence through learning capital (see Hypothesis 2), only learning capital should show incremental predictive power.

Several studies support our hypothesis. In a study by Harder et al. (2018), the capitals accounted for 19.2% of the variance in German language achievement, while intelligence explained 11.5%. In the same study, capitals and intelligence demonstrated approximately equal predictive power for mathematics achievement (R^2^ = 17.7% and 17.4%, respectively). In a study by Ziegler et al. (2019), capitals had incremental validity beyond IQ in predicting school achievement. The IQ test explained 23% of the variance. Adding the capitals increased the explained variance proportion to 37%. Paz-Baruch (2020) found that general intelligence, educational capital, and learning capital together accounted for 80% of the variance in scholastic achievements. However, learning capital made the highest individual contribution.

**Hypothesis** **2.** *Mediation Hypothesis.* The relationship between STEM educational capital and STEM achievement is mediated by STEM learning capital.

In a sense, educational capital represents the environmental potential. However, potential remains inert unless it is transformed into endogenous resources. A science book (infrastructural educational capital) does not improve grades. Only the knowledge and strategies acquired from it (actional learning capital) do. Such mediations have already been demonstrated in earlier studies by Veas et al. (2018) and Paz-Baruch (2020).

**Hypothesis** **3.** *Capital Interaction Hypothesis.* The interaction between educational capital and learning capital predicts STEM achievement.

An actiotope is a functional system. The interaction of its components produces outputs that go beyond their mere sum. A resource-rich environment has a greater impact the greater the utilization capacity, i.e., the learning capital. We therefore expect a synergistic effect where the interplay of both resources exceeds their additive effects.

**Hypothesis** **4.** *Intelligence Utilization Hypothesis.* General intelligence and STEM learning capital show a positive interaction effect on STEM achievement.

Since we interpret high intelligence as evidence of highly efficient resource utilization in the past, we expect this efficiency to be stable. Students who have successfully transformed resources before (i.e., who have a high IQ based on a history of success) should be more efficient at transforming their current STEM learning capital into academic achievement. This implies a “Matthew Effect”: Students with higher cognitive ability act as better “catalysts” for their resources, benefiting more strongly from the learning capital available to them (Ziegler & Stoeger, 2017b).

## 2. Method

### 2.1. Participants and Procedure

We analyzed data from 318 students (160 boys; *M*_age_ = 12.08, *SD*_age_ = 1.37) who were surveyed in autumn 2019 in secondary schools in Germany; 48% of them attended the highest-level secondary school in the German education system (Gymnasium), and 52% attended the middle-level secondary school (Realschule). Participants were in grades 6–10 and came from 42 classrooms in nine public schools in Germany.

The data collection was part of a project funded by the German Federal Ministry of Education and Research. It consisted of two surveys administered in schools using paper and pencil. First, a questionnaire assessed students’ educational and learning capital in STEM and their grades in STEM subjects. This questionnaire contained additional questions on students’ engagement with STEM and their beliefs about STEM learning, which were not analyzed in this study. Second, a matrix-based intelligence test was administered to assess general intelligence. The test administrators were teachers who received the test materials and instructions for conducting the surveys from the research team. After each survey, the teachers returned the completed questionnaires to the research team at the university, where student assistants entered the data into the computer.

The data was collected in pseudonymized form. To link the data from the student questionnaire and the intelligence test, each student was assigned an individual ID. The list assigning the IDs to students’ names was kept at the schools and was not accessible to the research team. Before data collection began, written consent was obtained from students and their parents. As the data was collected in schools, all questionnaires were reviewed and approved by the relevant German Ministry for Education and Cultural Affairs to ensure compliance with ethical standards and data protection regulations.

### 2.2. Measures

#### 2.2.1. Educational and Learning Capital in STEM

We assessed educational and learning capital in STEM using a shortened, adapted version of the Questionnaire of Educational and Learning Capital (Vladut et al., 2013). Each of the ten subscales of educational and learning capital was assessed using four items, each rated on a Likert-type scale from 1 (*completely disagree*) to 6 (*completely agree*). The educational capital in STEM and the learning capital in STEM were calculated by averaging the corresponding 20 items in the questionnaire. Cronbach’s alpha was 0.92 for educational capital in STEM and 0.95 for learning capital in STEM.

#### 2.2.2. General Intelligence

To measure general intelligence, we used three versions of the Hamdam Matrices Test covering different age groups: Students in grade 6 took the HMT 4–6 (Ziegler & Stoeger, 2016), students in grades 7 and 8 took the HMT 7–8 (Ziegler & Stoeger, 2017a), and students in grades 9 and 10 took the HMT 9–10 (Ziegler & Stoeger, 2018). The instrument is a nonverbal matrices-based intelligence test similar to the widely used Raven’s Progressive Matrices (Raven, 1938). Matrix-based intelligence tests have proven to be valid instruments to measure fluid intelligence, which is a core component of general intelligence (Carpenter et al., 1990). Each version consists of 28 items, each with four answer options. The time limit was set at 25 min.

As no German norms were available and our sample size was quite small, we followed a two-step procedure to obtain reliable norm-based test scores for general intelligence. First, the data from the three test versions were mapped using item linkage (71% overlap in items across test versions), and IRT-based person ability parameters were calculated for the whole sample. The marginal reliability of the latent ability estimate was 0.78. Second, we applied continuous norming, which produces more reliable results than conventional norming and is especially suitable for small sample sizes (A. Lenhard et al., 2018). To ensure sufficient sample sizes for calculating age-specific IQ scores, the norming was conducted across three age groups (10–11 years, 12–13 years, 14–15 years).

#### 2.2.3. STEM Achievement

Students reported their grades from their most recent report card in math, physics, chemistry, biology, and computer science. Self-reported grades are generally considered a reliable indicator of actual achievement (Sticca et al., 2017). In Germany, grades range from 1 (*excellent*) to 6 (*insufficient*). For our analysis, we reverse-coded students’ grades so that higher scores indicate higher achievement and calculated the mean.

### 2.3. Statistical Analysis

Analyses were conducted in the statistical environment of R (R Core Team, 2024) using the packages *mirt* (Chalmers, 2012), *cNORM* (W. Lenhard & Lenhard, 2023), *missForest* (Stekhoven, 2022), *robustbase* (Maechler et al., 2024), and *robmed* (Alfons & Ateş, 2022). Because six people had missing values in the STEM achievement variable, we imputed the data using random forest imputation (Stekhoven & Bühlmann, 2012). This machine-learning-based approach has proven robust and effective for imputing missing values across diverse scenarios. (Sun et al., 2023). In addition to the main study variables, we included gender, school type, grade level, and the first language spoken at home as auxiliary variables to improve the quality of missing-value imputation.

We tested our hypothesis using multiple linear regression analyses. Multicollinearity between predictor variables was assessed using VIF diagnostics. The VIF values ranged between 1.04 (STEM achievement) and 3.03 (learning capital in STEM), indicating no problematic multicollinearity. Since slight outliers were detected when testing regression assumptions, robust regressions were conducted using MM-estimation (Yohai, 1987). To evaluate the model fit, robust R^2^ was calculated. To make the parameter estimates for all predictors comparable and reduce multicollinearity when including interaction terms, we z-standardized IQ test scores, learning capital in STEM, and educational capital in STEM. Interaction terms were calculated using z-standardized variables.

To test the incremental predictive validity of students’ educational and learning capital in STEM and their interactive effect on students’ STEM achievement, we calculated a robust hierarchical regression. In the first step, we specified intelligence, educational capital in STEM, and learning capital in STEM as predictors. In the second step, we added the interaction term of educational and learning capital in STEM as an additional predictor. To test whether the relationship between students’ STEM educational capital and STEM achievement was mediated by their STEM learning capital, we conducted a robust mediation analysis. As suggested by Preacher and Hayes (2004), we used bootstrapping with 5000 resamples for the 95% bias-corrected confidence intervals to examine the indirect effects. Due to high collinearity between educational and learning capital in STEM, we tested the interaction between intelligence and learning capital in STEM in a separate robust regression, excluding educational capital in STEM. As we had clear expectations regarding the direction of the effects in testing our theory-based hypotheses, *p*-values in the main analysis were derived from one-sided significance tests in accordance with recommendations (Greenland et al., 2016). Since our analysis is based on cross-sectional data, no conclusions on causality regarding the relations between study variables can be drawn.

## 3. Results

### 3.1. Preliminary Analysis

Table 1 presents the means, standard deviations, and intercorrelations between the study variables. The inspection of histograms revealed that educational capital in STEM, learning capital in STEM, and general intelligence were normally distributed, with very few cases at the lower and upper ends of the scale. In contrast, performance in STEM subjects was slightly skewed to the right, with an accumulation of cases at the maximum value (19 people had a mean score of 6). Educational capital in STEM and learning capital in STEM were strongly intercorrelated and slightly correlated with general intelligence. All three predictors were moderately correlated with STEM achievement.

### 3.2. Main Analyses

In the robust hierarchical regression (see Table 2), students’ STEM achievement was predicted by intelligence and learning capital in STEM, but not by educational capital in STEM (Model 1). That supports our incremental validity hypothesis (H1), which posits that learning capital has an additional effect on students’ achievement beyond IQ. The inclusion of the interaction term between educational and learning capital in STEM (Model 2) additionally predicted students’ STEM achievement beyond intelligence and learning capital in STEM, supporting our capital interaction hypothesis (H3). The robust R^2^ were both moderate. They indicate that 18% of the variance in STEM achievement was explained by the predictors in Model 1, and 19% was explained by the predictors in Model 2.

**Figure 1:**
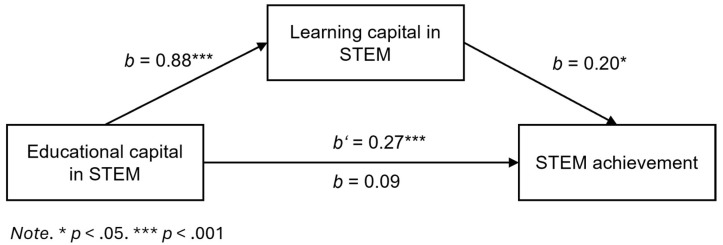
Robust Mediation Analysis (Hypothesis 2).

The results of the mediation analysis are illustrated in Figure 1. Students’ educational capital in STEM predicted both STEM learning capital (*b* = 0.88, SE = 0.03, *p* < .001) and STEM achievement (*b* = 0.27, SE = 0.05, *p* < .001). When including both capitals in the prediction of students’ STEM achievement, the effect of educational capital in STEM disappeared (*b* = 0.09, SE = 0.08, *p* = .132), while students’ learning capital in STEM remained as a significant predictor (*b* = 0.20, SE = 0.09, *p* = .011). This suggests full mediation with a bootstrapped indirect effect of *b* = 0.17, 95% CI [0.02, 0.32], supporting our mediation hypothesis (H2). The robust R^2^ for the full model was rather low, indicating that 12% of the variance in STEM achievement was explained by the predictors.

Table 3 contains the results for the robust regression on the interactive effect of intelligence and learning capital in STEM. As expected, the interaction term was a significant additional predictor, supporting our intelligence utilization hypothesis (H4) that learning capital can be better translated into actual achievement among students with higher intelligence. The robust R^2^ of this model was moderate, indicating an explained variance of 18%.

## 4. Discussion

The central aim of this study was to contribute to the search for broader models of talent development that extend beyond general intelligence (*g*). As emphasized throughout this Special Issue, high cognitive ability alone is not sufficient for exceptional performance. Much of the existing literature has responded to this gap by adding further traits—such as EQ or CQ—to the definition of giftedness (Matthews et al., 2018; Sternberg, 2005; Treffinger, 1986). Our approach takes a different direction. Rather than expanding the list of individual characteristics, we shift the focus to the systemic resources that enable talent development.

We assert a simple insight: wherever there are resources available and aligned, there is also talent. Our results provide empirical support for this view. Achievement emerges from how resources are organized and used, not solely from cognitive capacity.

Our first major finding confirms previous research that current resources matter beyond past cognitive history (Harder et al., 2018; Paz-Baruch, 2020; Ziegler et al., 2019). Learning capital in STEM predicted achievement over and above general intelligence (*g*) (Hypothesis 1). In our theoretical framework, we defined IQ not as a guarantee for the future, but as a measure of an effective action repertoire built in the past. The finding that learning capital explained unique variance suggests that high processing efficiency (IQ) alone is insufficient if the specific “programs” and “data” (current learning capital) are missing.

The next step in our analysis addressed the mechanism by which environmental resources affect achievement (as measured by grades). The mediation analysis revealed a full mediation effect. Educational capital did not directly predict achievement; its influence was entirely transmitted through learning capital, confirming Hypothesis 2 and earlier research (Paz-Baruch, 2020; Veas et al., 2018). Thus, environmental resources function more as a potential. They remain inert until they are actively converted into endogenous learning capital. Simply put, the environment provides the offer, but it is the learner’s acquired endogenous resources that generate the learning outcome.

Regarding Hypothesis 3, we found a meaningful interaction between educational and learning capital. This aligns with the systemic logic of the actiotope, which assumes that different forms of capital must work in concert for their full effect to emerge (Ziegler & Stoeger, 2017b).

Finally, the significant interaction between intelligence and learning capital (Hypothesis 4) provides a nuanced answer to the role of IQ in a resource-based model. Our results are consistent with the “Matthew Effect”: Students with higher general intelligence benefited significantly more from the available learning capital than their peers with lower cognitive ability. This finding is crucial for the integration of IQ into modern talent models. It suggests that intelligence functions as a catalyst. Students with a higher IQ—who, by our definition, have successfully utilized resources in the past to build a strong action repertoire—are more efficient in exploiting their current resources. They achieve greater learning gains from the same amount of capital. IQ and resources should therefore not be seen as competing explanations for outstanding achievements. Instead, higher cognitive ability appears to amplify the effects of available resources.

### 4.1. Beyond Additive Models of Giftedness: A Systemic Integration

Established multidimensional models—such as Gagné’s Differentiated Model of Giftedness and Talent (Gagné, 2020), Sternberg’s WICS model (Sternberg, 2005), Heller’s Munich Model (Heller et al., 2005), or Renzulli’s Three-Ring Conception (Renzulli, 1986)—long ago recognized that high cognitive ability (IQ) alone is insufficient to explain exceptional achievement. Their contribution was pivotal in opening the field to non-cognitive factors, whether conceptualized as catalysts, moderators, or specific traits like task commitment.

However, these expansions often followed an additive logic. To account for unexplained variance, lists of moderators were extended, or distinct types of intelligence (e.g., Gardner, 2000; Hedlund, 2020) were juxtaposed. Rather than continuously adding variables, the AMG proposes a systemic alternative. Rather than introducing new categories, it integrates these factors functionally as capitals. What established models describe as “environmental catalysts” or “moderators” are re-conceptualized here as educational capital and learning capital. These are not static trait-like or background factors. Rather, resources are subject to transformation and systemic constraints. This perspective builds on earlier models but reframes their components as parts of a functional system.

For instance, competencies typically ascribed to EQ—such as emotional regulation, empathy, and persistence—are functionally covered by the interplay of telic learning capital (which defines values and desired target states), actional learning capital (which provides the specific regulatory strategies to maintain or reach these states), and episodic learning capital (which provides a wealth of personal experiences). Similarly, creativity (CQ) does not necessarily require a separate intelligence construct. From a resource perspective, creative innovation results from high episodic learning capital (a rich database of diverse experiences and patterns) modulated by telic learning capital (for example, the desire to try something new), enabling novel recombinations executed through flexible actional learning capital.

### 4.2. Practical Implications

The current paradigm of gifted education is largely organized around the individual, their selection and support. However, if exceptional performance is the output of a functional system (the actiotope), our approach to educational practice must change fundamentally (Ziegler & Stoeger, 2017b). A systemic approach extends individual identification to systemic identification and shifts the focus from individual support to systemic design. Intelligence then loses its central position and becomes part of a systemic dynamic.

#### 4.2.1. Systemic Identification: Mapping Resources and Dynamics

If we cease to focus on selection as the primary objective of gifted identification, diagnostics can no longer be limited to a status check of the individual—whether IQ-based or achievement-based. Instead, it becomes an analysis of the functional relationship between the learner and their environment. “What configuration of resources is required to realize their learning potential?” becomes the central question. Diagnostics becomes the operative foundation of a Systemic Development Plan. This requires us to map the learner’s current profile of educational and learning capitals, their fit, and their dynamics.

*Resource Mapping:* The goal of resource mapping is to map a learner’s current profile of educational and learning capitals. It consists of three elements: identifying which resources are currently used, which are present but lying dormant, and which still need to be developed. For instance, a student may be highly motivated (telic learning capital) but still lack the targeted mentoring (social educational capital) needed to turn that motivation into high performance.

*Functional Alignment:* The goal of functional alignment is to identify a learner’s fit of educational and learning capitals; in other words, whether the environment provides the specific inputs required by the learner’s unique profile of educational and learning capitals, or if there is a mismatch that creates unnecessary friction. It is a resource-based assessment of whether learners are in the zone of proximal development, as defined by Vygotsky (1962).

*Developmental Dynamics:* The goal is to identify the developmental dynamics of a learner’s educational and learning capital. Instead of capturing a snapshot of the current state of learning and development, systemic diagnostics aims to provide the informational basis for designing a learning and development trajectory. For example, it asks: If we add a specific resource (e.g., a specific instructional strategy), how will the system respond? This turns diagnostics into a hypothesis-generating process for intervention, looking for the most effective lever to set the system in motion.

#### 4.2.2. The Intervention Shift: From Ad Hoc Support to Systemic Development Plans

Sustainable talent development requires moving from ad hoc support and compensation to Systemic Design. This is the function of the Systemic Development Plan (SDP). Unlike conventional support plans, an SDP focuses on aligning the environment to the learner’s profile. It operates on four design principles that apply to any trajectory of excellence.

*Access as Opportunity Structure:* Two central objectives of an SDP are to enrich an actiotope with resources and make it functionally accessible. It moves from a logic of provision (“what do we have?”) to a growth logic (“what does the talent require?”).

*Agency as Distributed Systemic Competence:* In an SDP, agency is not seen as a solitary trait of the learner but a functional property of the entire actiotope. In high-performance systems, regulation is shared among a network of actors—mentors, parents, teachers, and the student. The SDP orchestrates this “shared leadership”. It defines who carries the regulatory load at which developmental stage, ensuring that the necessary decisions and resource acquisitions are executed by the most capable agent in the network. The goal is to transform the social environment from a loose collection of supporters into a synchronized development team, within which the learner progressively assumes the role of the pilot.

*Action as Systemic Activation:* Even with sufficient resources and clear agency, development only occurs when these elements are brought into a specific functional interaction. The SDP aligns task demands and available capital into a coherent feedback loop. The goal is to ensure that the orchestrated resources are not just present but are effectively triggered to produce learning.

*Renewal as Systemic Sustainability:* Excellence consumes resources. A robust system requires built-in mechanisms for energy regeneration. The SDP identifies and integrates sources of Telic and Organismic recharge—environments or activities that do not deplete but generate motivation and vitality—ensuring that the pursuit of high performance remains sustainable over the long term.

Taken together, support based on SDPs and the four design principles change what we expect educators to do. They are not gatekeepers deciding who enters the domain of excellence. Instead, they are system architects responsible for building the infrastructure that makes excellence possible (Sternberg, 2020).

### 4.3. Limitations and Future Research

Our study has several limitations concerning design, measurement, and generalizability that should be considered when interpreting the results and which provide directions for future research. First, although our theoretical model postulates a causal chain, our data is cross-sectional. While our mediation analysis supports the plausibility of the assumed direction, high achievers might have retrospectively perceived their environment as richer (reverse causation), or reciprocal effects might exist over time. Longitudinal designs are needed to capture the actual transformation process of resources.

Second, except for the intelligence test, all measures relied on student self-reports. Therefore, future studies should consider objective data, such as classroom quality ratings or external observer ratings of available infrastructure.

Third, the distribution of grades was right-skewed (ceiling effect). This restriction of range usually makes it harder to detect significant predictors. The finding of robust effects despite this limitation supports the validity of the resource-based approach.

Fourth, we tested our model in the STEM domain because its cumulative nature makes it sensitive to resource bottlenecks. It remains an open question whether these systemic dependencies are equally strict in domains with less hierarchical knowledge structures. Future studies should investigate whether our results hold for other contexts. One interesting field to explore might be artistic domains, which require different abilities from those required in STEM. Further research could therefore expand the analysis and use the STEAM approach, which combines STEM fields with the arts (Boy, 2013).

Fifth, a theoretical limitation lies in the statistical modeling of systemic assumptions. Linear regression models can only approximate the constraints of a systemic bottleneck system. While our interaction analyses capture the synergistic nature of the actiotope, future research might employ nonlinear modeling techniques with larger datasets.

A final aspect that deserves attention in future research is gender when it comes to the influence of resources on talent development in STEM. There is substantial research on obstacles and barriers that hinder girls and women from developing their potential in STEM (Xie et al., 2015). Examining how these are related to and interact with available resources at different stages of talent development would add new insights to the query for how to best support females’ talent development in STEM (Mullet et al., 2017).

## 5. Conclusions

The results of this study support a reorientation in gifted education. For decades, the field has operated under a logic of “Gold Mining”—the search for a static, pre-existing trait (IQ) assumed to guarantee future success. Our findings regarding the incremental validity and the mediation role of capitals suggest that this view is insufficient.

While high general intelligence serves as a powerful catalyst—as demonstrated by the interaction effect in our data—it remains a historical measure of past resource utilization. Alone, it does not ensure future progress. To produce excellence, the paradigm must shift. STEM excellence is the emergent output of a well-resourced and well-regulated actiotope.

## Figures and Tables

**Table 1:** Means, Standard Deviations and Correlations for Study Variables.

Measures	*M*	*SD*	*Min*	*Max*	1	2	3	4
1. Educational capital in STEM	3.56	0.88	1.00	6.00	—			
2. Learning capital in STEM	3.68	0.91	1.00	6.00	0.81 ***	—		
3. General intelligence	100.00	14.93	59.05	140.95	0.14 *	0.19 ***	—	
4. STEM achievement	4.66	0.79	1.00	6.00	0.26 **	0.31 ***	0.31 ***	—

*Note.* * *p* < .05. ** *p* < .01. *** *p* < .001.

**Table 2:** Robust Hierarchical Regression Predicting STEM Achievement (Hypothesis 1 & 3).

Predictor	*b*	SE(*b*)	*t*	*p*
Model 1				
(Intercept)	4.69	0.04	113.80	<.001
General intelligence	0.22	0.04	5.26	<.001
Educational capital in STEM	0.09	0.07	1.26	.104
Learning capital in STEM	0.13	0.08	1.75	.040
Robust R^2^	0.18			
Model 2				
(Intercept)	4.65	0.05	99.99	<.001
General intelligence	0.22	0.04	5.27	<.001
Educational capital in STEM	0.09	0.07	1.24	.108
Learning capital in STEM	0.15	0.08	1.91	.028
Educational capital × learning capital	0.05	0.03	1.69	.046
Robust R^2^	0.19			

*Note.* Predictor variables are z-standardized. All *p*-values are based on one-sided testing.

**Table 3:** Robust Regression Predicting STEM Achievement (Hypothesis 4).

Predictor	*b*	SE(*b*)	*t*	*p*
(Intercept)	4.67	0.04	112.39	<.001
General intelligence	0.21	0.04	4.96	<.001
Learning capital in STEM	0.21	0.05	4.33	<.001
Intelligence × learning capital	0.09	0.05	1.81	.036
Robust R^2^	0.18			

*Note.* Predictor variables are z-standardized. All *p*-values are based on one-sided.

## Data Availability

The dataset analyzed during the current study and the relevant materials are available from the corresponding author on reasonable request.

## Abbreviations

The following abbreviations are used in this manuscript:

AMG	Actiotope Model of Giftedness
STEM	Science, Technology, Engineering, Mathematics
